# Feasibility and benefits of home initiation of subcutaneous apomorphine infusion for patients with Parkinson’s disease: the APOKADO study

**DOI:** 10.1007/s00702-023-02609-6

**Published:** 2023-03-02

**Authors:** Fabien Zagnoli, Amélie Leblanc, Irina Viakhireva-Dovganyuk, Jean-Philippe Delabrousse-Mayoux, Alain Pouyet, Marc Ziegler, Laura Sogni, Marie Patat, Régis Bouillot, Marc Vérin, The APOKADO Group, Andrei Arhire, Philippe Barres, Maxime Blondiaux, Jean-Claude Bouffeteau, Jean-Philippe Brandel, Christophe Carel, Giovanni Castelnovo, Marc Coustans, Lucie Courault, Christian Crauser, Isabelle Degaey, Bertrand Degos, Jean-Philippe Delabrousse, Béatrice Denis, Marie-Claude Dourneau, Arnaud Duretete, Jean-Marc François Feve, Erika Follin, Michel Gugenheim, Cécile Hubsch, Nathalie Patte Karsenti, Pierre Louchart, Serge Massengo, José Mejias, Homero Monteiro, Philippe Muh, Bernard Pedespan, Virginie Sattler, Mathieu Sevin, Mélissa Tir, Anne Tirel Badets, Marc Verin, Irina Viakhireva, Elisabeth Vidry, Jean-Charles Wiart

**Affiliations:** 1 Neurology Office, 22 rue d’Aiguillon, 29200 Brest, France; 2 Neurology Department, Cavale Blanche University Hospital, Boulevard Tanguy Prigent, 29200 Brest, France; 3 Neurology Office, MSP Avenue de la Roque, 24100 Creysse, France; 4 Neurology Office, 3 Boulevard Waldeck Rousseau, 22000 Saint-Brieuc, France; 5 James Parkinson Unit, Rothschild Foundation, 29 Rue Manin, 75019 Paris, France; 6 Adelia Medical, 125 avenue Louis Roche, 92036 Gennevilliers, France; 7 Neurology Department, Pontchaillou University Hospital, rue Henri Le Guilloux, 35000 Rennes, France; 8 Institut of Clinical Neurosciences of Rennes, Rennes, France; 9 Behavior and Basal Ganglia Research Unit, CIC-IT INSERM, 1414 & University of Rennes, Rennes, France

**Keywords:** Parkinson’s disease, Apomorphine, Home care, Quality of life

## Abstract

Continuous subcutaneous apomorphine infusion (CSAI) is used to treat patients with Parkinson’s disease (PD) who are experiencing motor fluctuations. However, the need to initiate this treatment during a hospital stay may restrict patients’ access to it. To assess the feasibility and benefits of initiating CSAI in the patient’s own home. A French prospective multicenter longitudinal observational study (APOKADO) among patients with PD who required subcutaneous apomorphine, comparing in-hospital versus home initiation. Clinical status was assessed according to the Hoehn and Yahr score), the Unified Parkinson’s Disease Rating Scale Part III, and the Montreal Cognitive Assessment. We assessed patients’ quality of life with the 8-item Parkinson’s Disease Questionnaire, rated the improvement in their clinical status on the 7-point Clinical Global Impression–Improvement scale, recorded adverse events, and ran a cost–benefit analysis. 145 patients with motor fluctuations were included in 29 centers (office and hospital). Of these, 106 (74%) were initiated onto CSAI at home, and 38 (26%) in hospital. At inclusion, the two groups were comparable for all demographic and PD characteristics. After 6 months, quality of life, adverse events and early dropout rates were similarly rare-across the two groups. Patients in the home group improved more quickly their quality of life and became more autonomous in managing the device than those in the hospital group, and their care costed less. This study shows that home (versus in-hospital) initiation of CSAI is feasible, improves patients’ quality of life more quickly, with the same level of tolerance. It is also less expensive. This finding should make it easier for patients to access this treatment in the future.

**Supplementary Information:**

The online version contains supplementary material available at 10.1007/s00702-023-02609-6.

## Introduction

Apomorphine has been used as a treatment for Parkinson’s disease (PD) since the 1980s, as it acts on both D1 and D2 receptors, as well as on the nigrostriatal pathway, making it more similar to levodopa in terms of action and tolerance than most other dopamine agonists (Ribarič 2012; Auffret et al. 2018). It is quick acting, but has to be given as a subcutaneous injection. For the past 20 years, it has been possible to administer it either continuously, using an infusion pump, or intermittently, using an injector pen (Drapier and Vérin 2006). Extensive research in this area has yielded a fairly accurate picture of the indications and benefits of apomorphine, in terms of motor function and quality of life (Martinez-Martin et al. 2011; Grandas 2013; Borgemeester et al. 2016; Rosa-Grilo et al. 2016; Meira et al. 2021).

Up to now, initiation of continuous subcutaneous apomorphine infusion (CSAI) has required a hospital stay, generally in a PD specialist center or a hospital department with experience of the disease. Patients stay in hospital for between 5 and 10 days (Grandas 2013; Bhidayasiri et al. 2015; Trenkwalder et al. 2015; Katzenschlager et al. 2018) in order to enable clinicians to adjust the flow rate on a daily basis, modify patients’ oral medication, and look out for potential adverse effects. The hospital stay also gives patients and their caregivers an opportunity to familiarize themselves with the treatment. Once they have returned home, patients receive care from a team of local district nurses (Bhidayasiri et al. 2015) trained by the medical device supplier, who is responsible for the technical follow-up. They are monitored either by the neurologist who initiated the CSAI, or by their treating neurologist.

Over time, a number of medical, social and geographical drawbacks have become apparent (Henriksen et al. 2020). Some patients who could benefit from CSAI are put off by the wait for an appointment and the length of the subsequent hospital stay, as well as the physical distance from the nearest specialist center. The recent Covid pandemic episode also demonstrated the limits of hospital-based care when hospitals are no longer accessible (Afraie et al. 2022). As recently suggested, neurologists may be reluctant to prescribe a treatment if they cannot initiate themselves and to become involved in the follow-up (Fujioka et al. 2023). Last, the cost of the hospital stay comes on top of the cost of initiation and follow-up (Valldeoriola et al. 2013; Walter and Odin 2015).

Home initiation would overcome these drawbacks and allow more patients to access CSAI.

So far, most studies have focused on in-hospital initiation, but a recent Expert Consensus Group report indicated that it should be possible to initiate patients onto therapy at home, providing the team has the necessary experience (Castaño et al. 2007; Trenkwalder et al. 2015). We nevertheless need to confirm that home initiation is equivalent to in-hospital initiation in terms of efficacy, tolerance, and quality of life.

The aim of the present study was therefore to demonstrate the feasibility of home initiation, and to compare the two modalities on clinical efficacy, tolerance, improvement in quality of life, and cost.

## Materials and methods

The APOKADO study was a French prospective multicenter longitudinal observational nonrandomized study approved by the CCP Ile de France V institutional review board (18.07.16.4828 CAT3, 3 August 2016). It included patients who had been diagnosed with PD at least 5 years earlier and who had an indication for apomorphine according to their neurologist. The eligible patients were from early fluctuators (Fernández-Pajarín et al. 2022) to advanced PD (Drapier et al. 2016). We excluded patients with an atypical Parkinsonian syndrome, as well as those exhibiting cognitive decline or severe psychotic disorders, or who were unable to complete the self-report questionnaires. All the patients included in the study took part on a voluntary basis and signed an informed consent form.

Patients were assigned to one of two groups (hospital or home), depending on whether their neurologist worked: at a hospital (hospital or home group) or in a private practice (home group). The decision on where to start was made by the neurologist.

Patients’ clinical status when they joined the study (Day 0, D0) was assessed by the investigating neurologist according to the Hoehn and Yahr score (Hoehn and Yahr 1967), the Unified Parkinson’s Disease Rating Scale Part III (UPDRS-III) (Ramaker et al. 2002), and the Montreal Cognitive Assessment (MoCA) (Dalrymple-Alford et al. 2010). A quality of life scale (8-item Parkinson's Disease Questionnaire, PDQ-8) (Jenkinson et al. 1998)) and a scale measuring the ability to perform everyday activities (Instrumental Activities of Daily Living, IADL) were completed by the patient and/or informal caregiver (Lawton and Brody 1969).

Titration of the apomorphine flow and adaptation of the oral treatment was progressive according to the patient's clinical condition. Patients were educated for home treatment by the nurses of the medical device supplier.

Patients were seen by the investigating neurologist at 1 month (M1), 3 months (M3), and 6 months (M6) post-initiation. On each occasion, the investigator assessed their clinical status, the Hoehn and Yahr score and recorded any adverse events. Patients completed the PDQ-8 and a self-report questionnaire created especially for this study to assess their autonomy in managing their treatment. The Clinical Global Impression—Improvement scale (CGI-I) assessing patients’ clinical improvement on a 7-point scale (*very much improved*, *much improved*, *minimally improved*, *no change*, *minimally worse*, *much worse*, and *very much worse*) (Drapier et al. 2016; Guy 1976) was completed by both the patient and the investigating neurologist at M1, M3, and M6. Last, we calculated the number of days spent in hospital, and the numbers of patient transport journeys, medical consultations, and visits by district nurses and the medical device supplier (see Supplementary Material).

The criteria for the analysis were changes in patients’ perceived quality of life and their clinical status, assessed by both patient and investigator, as well as the occurrence of adverse effects, patients’ autonomy in managing their treatment, and the estimated cost of the treatment over the 6-month follow-up period. For quality of life, we analyzed the PDQ-8 scores at each timepoint (continuous variable), as well as the percentages of patients with a better, poorer or stable quality of life (categorical variable). More specifically, we deemed quality of life to have improved if the PDQ-8 score fell by at least 5.94 points, compared with baseline (D0), and to have worsened if this score rose by at least 4.91 points. Between the two, we considered quality of life to be stable (Horváth et al. 2017). To calculate the cost of CSAI initiation, we obtained the prices (2021) of the different healthcare services and procedures from the National Health Insurance Fund (Table 2).

First, for each continuous variable, we computed the mean value and standard deviation (*SD*), the minimum and maximum values, the median, and the interquartile range (Q1–Q3). For each categorical variable, we calculated the number of patients and the percentage (%). Next, for the continuous variables, after we had checked the normality of the distribution (Shapiro–Wilk test), we ran group comparisons using either the Student *t* test (normal distribution) or the nonparametric Kruskal–Wallis test (non-normal distribution). For the categorical variables, we performed comparisons with the chi-square or Fisher test when the numbers and percentages were sufficiently high. For quality of life (PDQ-8 score; continuous variable), we ran an analysis of variance (ANOVA) to assess the effects of time and initiation modality (home vs. in-hospital), and the possible interactions between the two. To compare any improvements in quality of life (PDQ-8 score; categorical variable) and clinical status (CGI-I) over the 6-month follow-up, we ran a chi^2^ test to assess the effect of modality, and Cochran’s Q test to assess the effect of time. The significance threshold was set at 0.05 for all these tests.

According to the study protocol, we needed 64 patients in each group (i.e. total of 128 patients) in order to detect a meaningful difference between the two groups (≥ 0.5 × *SD*), corresponding to a medium effect size, with an alpha risk of 5% and power of 80%.

### Role of the funding source

The funder of the study (Adelia Medical) had no role in study design, data collection, data analysis, data interpretation, or writing of the report.

## Results

A total of 145 patients (84 men and 61 women), with a mean age of 70.1 years (range: 37–92) were included between 17 September 2018 and 30 December 2020. Mean disease duration was 11.1 years (range: 2–30). The mean Hoehn and Yahr score was 2.2 (range: 0–5) on medication, and 2.8 (1–5) off medication. The mean UPDRS-III score off medication was 35.1 (8–68). The mean MoCA score was 25/30 (range: 6–30). The mean IADL score was 1.35 (0–4), and the mean baseline PDQ-8 score was 39.6 (0–69). A total of 80% of patients had an informal caregiver, 8.6% were in employment, and 17.1% were registered as having a disability because of their disease. A total of 40% had at least a high-school diploma. CSAI was indicated for motor fluctuations (91% of patients), gait problems (29%), difficulty swallowing (4.8%), or as a stop-gap whilst awaiting deep-brain stimulation (2.1%). Patients could have more than one indication.

A total of 44 neurologists from 32 different centers took part in the present study: 19 were working at hospital (10 in a Parkinson expert center and 9 in department of neurology), and 25 in private practice. As a result, 106 patients were initiated on CSAI at home, and 38 in hospital (data were missing for one patient, who was excluded from group analyses). The home and hospital groups were similar on age, sex ratio, disease duration, motor symptoms (UPDRS-III, Hoehn and Yahr), cognitive status (MoCA), ability to perform everyday activities (IADL), quality of life (PDQ-8), CSAI indication, presence of an informal caregiver, and socioeconomic status (Table 1). They were also similar on education level and medical history (data not shown).

**Table 1:** Baseline Characteristics of Patients in Each Group

	All patients *N* = 144	Home *n* = 106	Hospital *n* = 38	*p* value
Mean age in years (*SD*)	70.1 (9.1)	69.9 (9.3)	70.5(8.8)	0.74
Men: *n* (%)	84 (57.9)	64 (60.4)	20 (52.6)	0.52
Mean disease duration in years (*SD*)	11.1 (5.4)	11.1 (5.8)	11.3 (4.2)	0.26
Mean UPDRS-III OFF score (*SD*)	35.1 (17.8)	33.9 (18.0)	37.3 (17.1)	0.67
Mean Hoehn & Yahr ON score (*SD*)	2.2 (0.9)	2.2 (0.9)	2.3 (0.9)	0.63
Mean MoCA score (*SD*)	25 (3.7)	24.8 (4.1)	25.6 (2.9)	0.52
Mean IADL score (*SD*)	1.35 (1.44)	1.3 (1.4)	1.5 (1.5)	0.44
Mean PDQ-8 score (*SD*)	39.6 (1.48)	39 (14.7)	41 (15.1)	0.53
Informal caregiver: *n* (%)	116 (80)	89 (84.0)	27 (71.0)	0.14
Currently in employment: *n* (%)	12 (8.27)	10 (9.88)	2 (5.6)	0.73
Disabling motor fluctuations: *n* (%)	132 (91)	98 (92.5)	34 (89.5)	0.52
Mean LED/d	1285 (386)	1304 (384)	1252 (399)	*0.33*

Missing data were not replaced. Tests: Student *t*, chi^2^, or Kruskal–Wallis

*IADL* Instrumental Activities of Daily Living, *MoCA* Montreal Cognitive Assessment, *PDQ-8* 8-item Parkinson's Disease Questionnaire, *SD* standard deviation, *UPDRS-III* Unified Parkinson’s Disease Rating Scale Part III, *LED* levodopa equivalent dose

Apomorphine (Aguettant Pharma (Lyon, France)) was delivered subcutaneously by a pump (CRONO PAR, Pentaferte France, Villeparisis, France). Participants started treatment at home under the technical supervision of a home health-care professional (provided by Adelia Medical, Gennevilliers, France).

### Treatment progress

If during the study there was no difference in LED between the two groups at inclusion and then at M1, M3, and M6, nor any significant difference in the duration of infusion (15h30/day on average at M1 and 18 h/day at M6), on the other hand, it was observed that in the hospital group apomorphine titration was faster and the proportion of this treatment was greater, representing 47% *(SD* = *23,2)* at M1, 50.3% *(SD* = *22,6)* at M3 and 52.3% *(SD* = *22,7)* at M6, compared with 31% *(SD* = *14)* at M1, 38.8% *(SD* = *16,5)* at M3 and 43.8% *(SD* = *19,3)* at M6, respectively in the home group. This difference was significant at M1 (*p* = 0.001) and M3 (*p* = 0.029) but not at M6 (*p* = 0.09).

### Quality of life

**Fig. 1:**
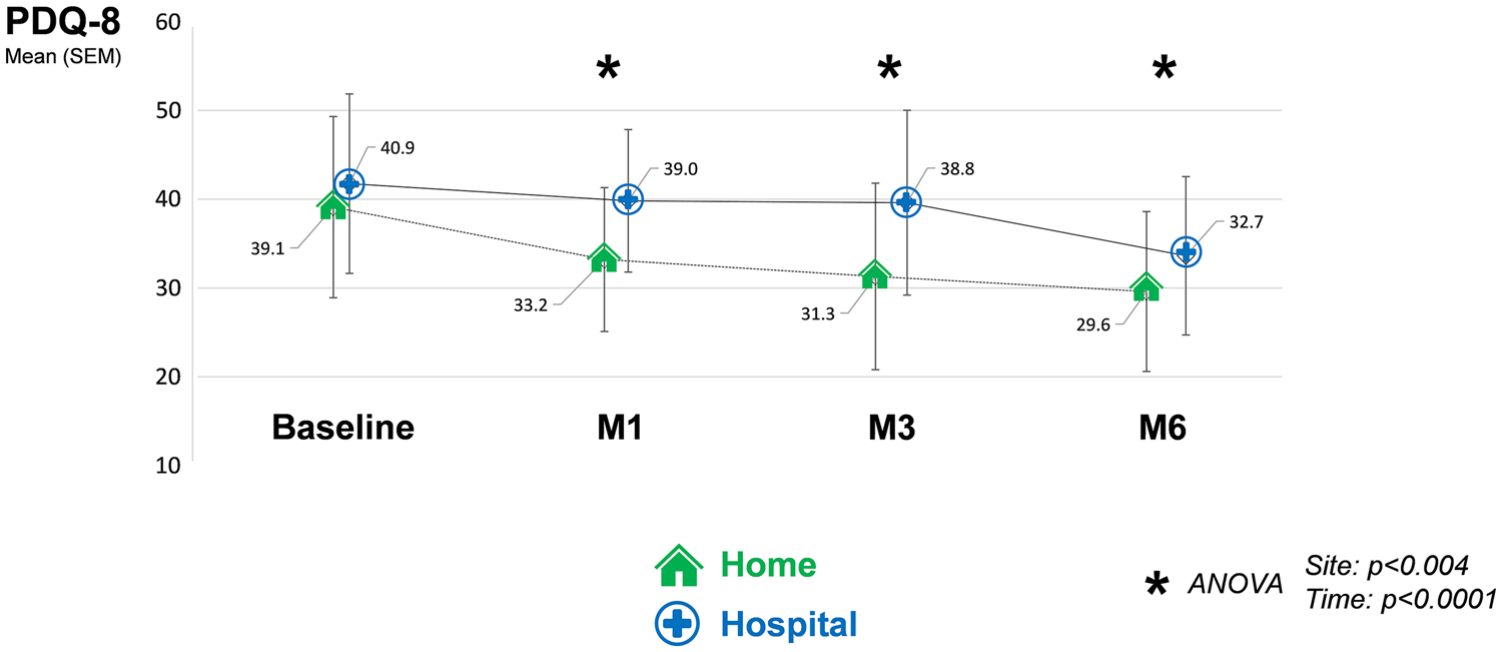
Changes in PDQ-8 scores during the first 6 months of CSAI according to initiation modality: home (*n* = 106) vs. in-hospital (*n* = 38)

PDQ-8 scores fell over time (Fig. 1), reflecting an improvement in patients’ quality of life. For both groups, these scores fell significantly between CSAI initiation (baseline) and the final assessment at M6 (ANOVA, *p* < 0.0001). However, there was a significant improvement as early as the first month of treatment (ANOVA, *p* < 0.004) in the home group in comparison with the hospital group, with a difference of 4.5 points (10%) in the PDQ-8 score between the two groups, in favor of the home one.

**Fig. 2:**
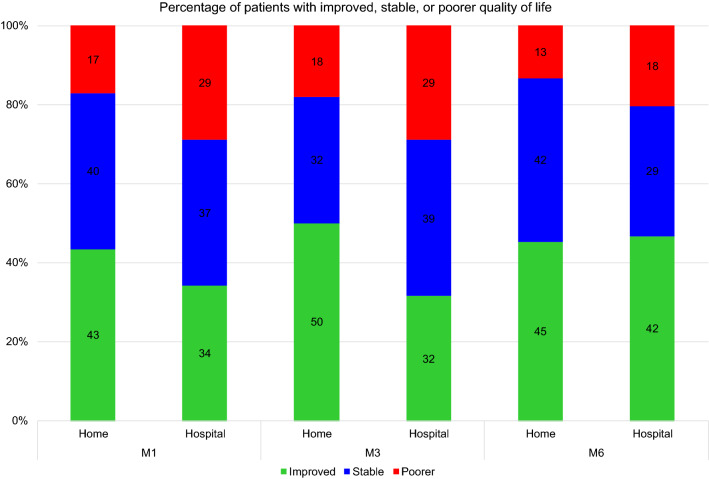
Treatment response as reflected in improved quality of life according to time and CSAI initiation modality

The percentage of patients whose quality of life improved after CSAI initiation (reduction in PDQ-8 score of more than 5.94 points) was significantly higher in the home group than in the hospital group (*p* = 0.048). This percentage varied over time as follows: between 43% at M1 and 50% at M3 for the home group, and between 34% at M1 and 42% at M3 for the hospital group. There were fewer patients with a reduced quality of life (increase in PDQ-8 score of more than 4.91 points) in the home group (13–18%) than in the hospital group (18–29%) (Fig. 2).

### Clinical improvement

**Fig. 3:**
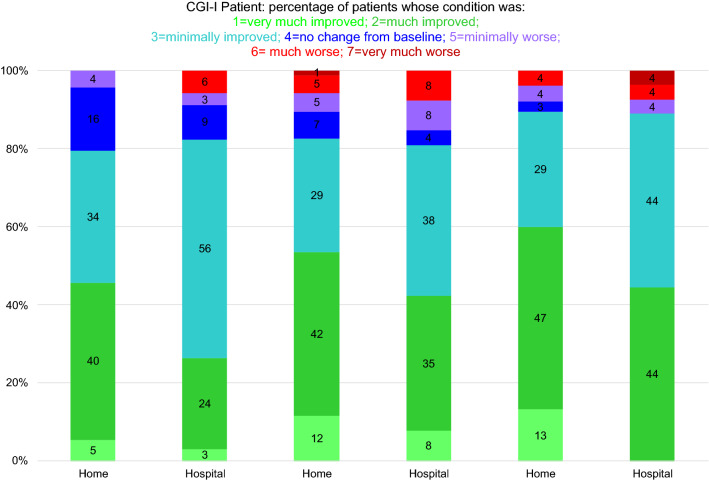
Distribution of patients according to their clinical status (patient ratings) 1, 3, and 6 months after home (*n* = 106) or in-hospital (*n* = 38) CSAI initiation

**Fig. 4:**
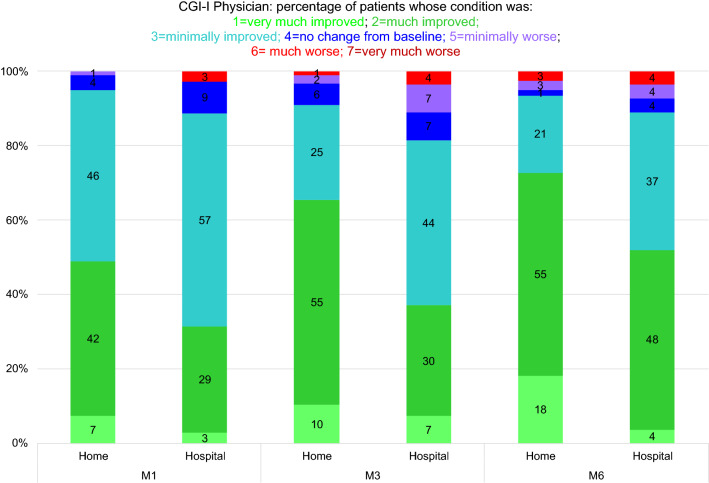
Distribution of patients according to their clinical status (investigator ratings) 1, 3, and 6 months after home (*n* = 106) or in-hospital (*n* = 38) CSAI initiation

As early as the first month, many patients experienced an improvement in their clinical status, from both their own point of view and that of the investigator, particularly in the home group. The percentages of patients whose clinical status was *much improved* or *very much improved* remained stable and even increased over time (Figs. 3, 4).

The distribution of patients across the seven CGI-I categories (patient ratings) differed significantly between groups (*p* = 0.023). At every timepoint, the percentages of patients who reported that their status was *much improved* or *very much improved* were significantly higher in the home group than in the hospital group (Fig. 3). The results were the same for the investigator ratings (*p* = 0.010) (Fig. 4).

The distribution of patients across the CGI-I categories according to the investigator ratings varied over time (*p* < 0.001). The number of patients whose clinical status was perceived by the investigator to have only *minimally improved* was higher at M1 than at M3 and M6, whereas the number of patients whose clinical status was perceived to be *much improved* or *very much improved* was higher at M6 than at M1 and M3 (Fig. 4). Although the distribution of patients across the CGI-I categories according to the patient ratings did not differ significantly, the *p* value (0.067) was close to the significance threshold (0.05), and the percentages of patients who reported that their status was *much improved* or *very much improved* tended to increase after M1 in both groups (Figs. 3, 4).

### Autonomy

**Fig. 5:**
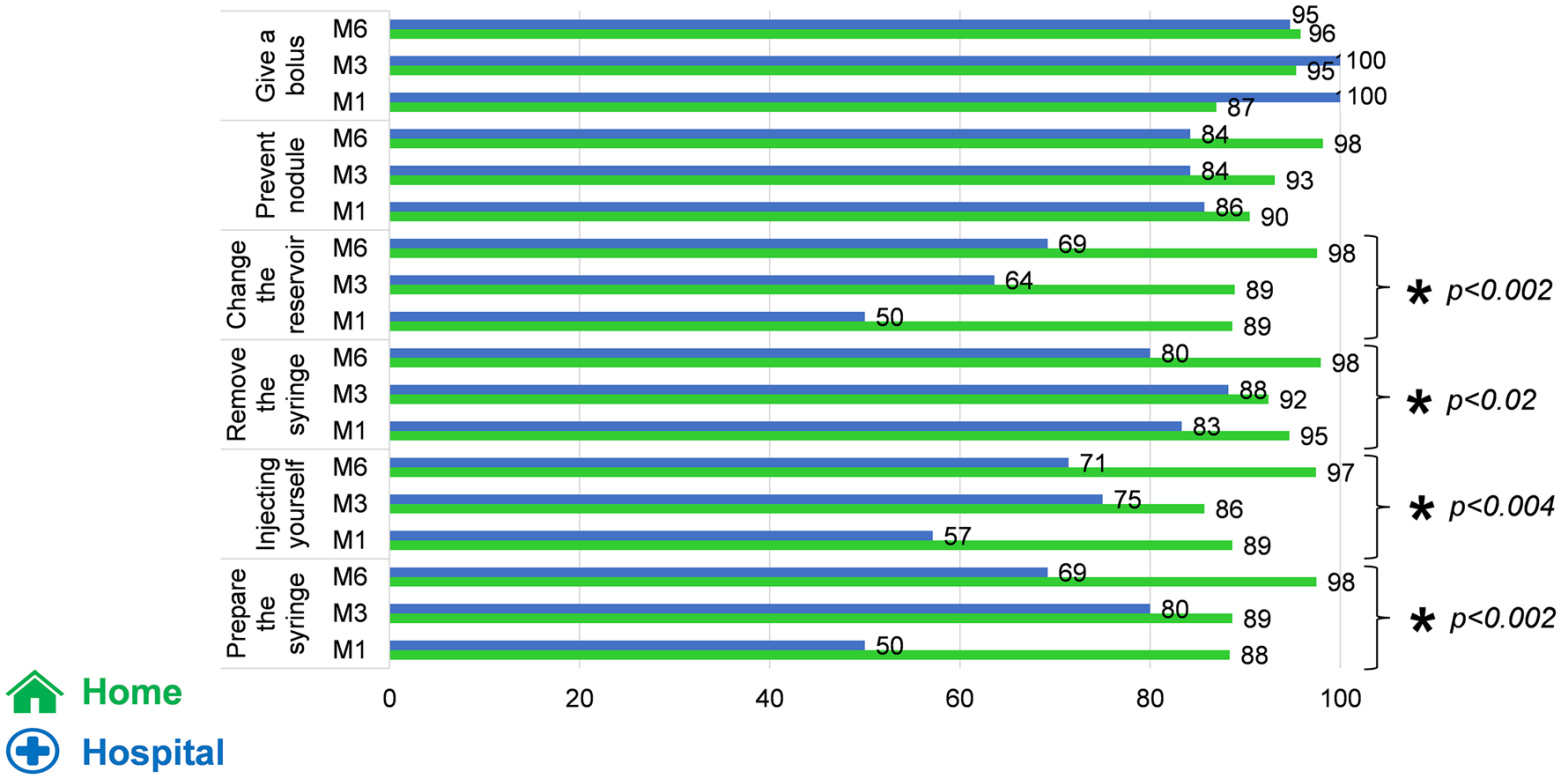
Percentages of patients reporting autonomy in managing their treatment

At M1, M3, and M6, for most of the items (change the reservoir, remove the syringe, injecting yourself, prepare the syringe), significantly more patients in the home group reported that they had control over their treatment and were autonomous in its management in comparison with the patients of the hospital group (Fig. 5).

### Side effects and dropouts

For all patients, bruising or itching at the injection site was reported by around 25% of patients, and small nodules by 33% of them, regardless of initiation modality. There was a single reported case of skin necrosis in each group.

Despite the utilization of domperidone (30 mg/d during the two first weeks and then decrease and stop), nausea was reported by at least 20% of patients at M1 and 10% at M6, but this did not affect the prescribing of apomorphine. Episodes of mental confusion were reported in around 10% of patients across follow-up, and light to moderate hallucinations in 20% of patients at M1 and 26.4% at M6. Only three patients, all in the home group, exhibited severe forms of hallucinations, and these regressed once their treatment had been adjusted (stop dopaminergic agonist and addition of clozapine: 12,5 to 25 mg/d for the three and decrease of 10% to 20% daily apomorphine for two of them). Behavioral disorders were reported in 5.3% cases at M1. These were light to moderate, and did not worsen over time. Orthostatic hypotension was found in 16.7% of patients. This only warranted corrective treatment in five cases (four in the home group, and one in the hospital group). Light or moderate dyskinesias were noted in approximately 25% of cases, probably due to insufficiently high apomorphine flow for some patients, associated with insufficient reduction of oral dopaminergic therapy, as usually reported (García Ruiz et al. 2008). However, only two of which (both in the home group) were described as *severe*.

**Fig. 6:**
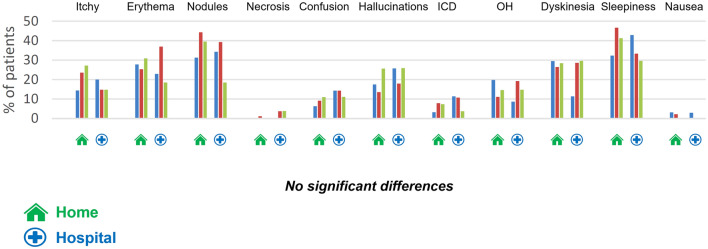
Percentage of patients reporting a side effect in each group at M1, M3 and M6. *ICD* impulse control disorders, *OH* orthostatic hypotension, Skin: itchy, erythema, nodules and necrosis

The frequency of side effects was similar regardless of initiation modality (Fig. 6). Most of these side effects were light to moderate, while out of the 34 severe side effects we recorded, 23 were reported in the home group, and 11 in the hospital group. In most cases, they took the form of sleepiness (seven cases in the home group, and six in the hospital one). There were four cases of hypotension and three of nausea in the home group.

Altogether, there were 22 dropouts (i.e., 15.3% of patients): 17 (16%) in the home group, and five (13%) in the hospital one. Eight occurred within the first month of treatment. Ten (7%), including seven (6.6%) in the home group and three (7.8%) in the hospital group, were due to a loss of motivation when confronted with the challenges of using the device. Three were due to difficulty controlling impulses, and three to the onset of mental confusion. All of these occurred within the first few weeks of treatment. A single patient had to stop the treatment owing to severe orthostatic hypotension.

Last, there were four deaths, three following lung infections that occurred shortly after CSAI initiation in patients who had started the treatment because of severe difficulty swallowing.

### Cost analysis

On average, the financial cost per person for the first month following home initiation comprised 1.6 neurology consultations, 7.4 visits by the medical device supplier, and 42 visits by a district nurse. During this same month, patients in the hospital group spent an average of 7.3 days in hospital. On average, there were 5.1 visits by the device supplier, and 30 by the district nurse. Beyond M1, on average, there were slightly more medical consultations and slightly fewer visits by district nurses for the home group than for the hospital one, as well as fewer days in hospital.

Across the 6-month follow-up, patients in the home (versus hospital) group required more medical consultations (3.4 vs. 1.5) and visits by district nurses (114 vs. 75), but virtually the same number of patient transport journeys (2.2 vs. 2.5), and they crucially avoided 10 hospital inpatient days (0.9 vs. 10.9). As the medical device supplier charged a monthly fee based on the number of days, this fee was lower for the hospital group than for the home one at M1, but the same across the two groups in all subsequent months. The mean difference in cost between the two modalities was therefore estimated at €11 387 per patient (Table 2).

**Table 2:** Costs (in Euros) Generated by CSAI Initiation According to Modality

Item	Unit cost	Period	Home × *n* = €	Hospital × *n* = €
Hospital inpatient days (initial stay + subsequent stay)	€1370.00	M1	× 0.9 = €1233	× 10.9 = €14 933
Daily cost of treatment	€ 15	D0 to M6	× 180 = €2700	× 180 = €2700
Neurology consultations	€46.70	D0 to M6	× 3.4 = €158.78	× 1.5 = €70.05
Patient transport journeys	€50	D0 to M6	× 2.2 = €110	× 2.5 = €125
Initiation fee	€297	M1	× 1 = €297	× 1 = €297
Medical device supplier fee for M1	€1158	M1	× 1 = €1158	× 0.75 = €869.50
Monthly device supplier fee	€1158	M1 to M6	× 5 = €5790	× 5 = €5790
Visits by district nurse	€50	D0 to M6	× 114 = €5700	× 75 = €3750
Total			€17 146,78	€28 533,55
Difference			€11 386.77	

*Source*: French National Health Insurance 2021

## Discussion

Our sample was slightly older (*M*_age_ = 70 years) than those in most other studies (Drapier et al. 2016; Kimber et al. 2017; Sesar et al. 2017; Dafsari et al. 2019; Meira et al. 2021), but similar in terms of disease duration (Drapier et al. 2016; Katzenschlager et al. 2021; Phokaewvarangkul et al. 2021), clinical status (as assessed with Hoehn & Yahr and UPDRS-III) (Drapier et al. 2016; Dafsari et al. 2019; Phokaewvarangkul et al. 2021), and quality of life (as assessed with the PDQ-8 Martinez-Martin et al. 2015; Dafsari et al. 2019) or PDQ-39 (Drapier et al. 2016; Houvenaghel et al. 2018) to those in other studies assessing the efficacy of CSAI. For 91% of participants in our study, the motivation for initiating this therapy was the presence of motor fluctuations, as was the case in most studies (Drapier et al. 2016; Borgemeester and van Laar 2017; Katzenschlager et al. 2018). Moreover, as elsewhere, 80% of our patients had an informal caregiver (Grandas 2013; Phokaewvarangkul et al. 2021). Given that our two groups (i.e., home and hospital) did not differ on any of the above characteristics, we were able to run intergroup comparisons.

Although several studies did not objectively measure the improvement in quality of life, using either the PDQ-8 (Katzenschlager et al. 2021) or PDQ-39 (Houvenaghel et al. 2018), many others did do so, reporting mean improvements of between 11 and 42% (Martinez-Martin et al. 2011, 2015; Drapier et al. 2016; Dafsari et al. 2019; Phokaewvarangkul et al. 2021; Fernández-Pajarín et al. 2022), particularly during the first 6–12 months of CSAI (Meira et al. 2021). In our study, quality of life as measured with the PDQ-8 had improved by an average of 9.2 points (i.e. 21%) after 6 months of treatment across the whole sample, and by 13.7 points (= 9.2 + 4.5) points (i.e. 32%) for patients in the home group. Moreover, this improvement generally happened sooner in the home group than in the hospital group, with 43.4% of patients reporting an improved quality of life (reduction of 5.94 points or more in the PDQ-8 score) (Horváth et al. 2017) as early as M1, compared with 34.2% in the hospital group. It should be noted that the mean improvement at M1 was 9.4 points for the home group versus 4.7 points for the hospital group.

Some studies (Kimber et al. 2017; Katzenschlager et al. 2021) that also considered the CGI-I found that more than 70% of patients improved after commencing CSAI. However, when we looked solely at patients whose clinical status had either *much* or *very much improved*, this proportion was a more modest 30% after 3 months of treatment(Katzenschlager et al. 2018), rising to between 45 and 66% after 6 months (Drapier and Vérin 2006; Houvenaghel et al. 2018). These figures are similar to those we found for our participants, and reflect the gradual nature of the clinical improvement and the value of maintaining the treatment for several months at least, to properly gauge its usefulness.

We observed a significant difference according to the modality of initiation as follows: half the patients in the home group stated that their clinical status had *much* or *very much improved* after the first month of treatment, compared with just a third in the hospital group.

The PDQ-8 and CGI-I therefore highlighted improvements in most patients, comparable to those reported in the literature, but these improvements took place sooner among patients in the home group.

This faster improvement in quality of life and more marked improvement in clinical status is probably not unequivocal. As our two groups have similar characteristics, the lack of randomization does not seem to explain this difference. The fact that the titration of apomorphine and the reduction of the oral treatment are more progressive in the home group could explained a better comfort felt by the patient even though the total doses of LED are similar (Maricle et al. 1995; Rabinak and Nirenberg 2010). This may be a pharmacological effect and/or a psychological dimension of a more gradual therapeutic change.

Although the impact of using nurse specialists is still difficult to demonstrate (Hagell 2007), this swifter and stronger improvement in our study probably reflects the importance of personalized care delivered to patients in their own home by the medical device supplier and team of district nurses. Regular visits by a single specialist supplier who is prepared to spend time with the patient and caregiver ensure continuity of care in the initial phase and install a climate of confidence that reinforces treatment adherence. Daily visits from district nurses, often already known to the patients and who could spot side effects in a timely fashion are another reassuring factor-and a source of patient and caregiver satisfaction (Reynolds et al. 2000; Jarman et al. 2002; Tan et al. 2014; Roszmann et al. 2022). We can, therefore, conclude that this personalized approach has a genuine impact on patients’ assessment of the care they receive and on the degree of improvement in their clinical status and quality of life. This underlines the fact that the success of a given treatment depends not only on the choice of medication, but also on the extent to which the patient (and caregiver, where relevant) understands the disease and is able to manage the device (Bhidayasiri et al. 2016), and the latter is enhanced when assistance is given in the patient’s own home (Jahanshahi et al. 1994; Tan et al. 2014).

For some patients, being able to insert the needle and set up the device themselves may be an additional convenience (fewer home visits from healthcare professionals, easier to travel and go on holiday) but autonomy is not an end in itself, and frailer patients may find daily visits by a district nurse reassuring (Bloem et al. 2020). In our study, patients had generally acquired a degree of autonomy by the end of the first month of treatment, particularly when it came to changing the syringe and inserting the needle.

Similar proportions of mild to moderate side effects were found in each group. Problematic subcutaneous nodules were reported by more than a third of patients in our study, which is equivalent to the proportions reported in other recent studies (44%) (Katzenschlager et al. 2018, 2021), and lower than those reported in earlier ones (Deleu et al. 2004; García Ruiz et al. 2008) probably reflecting an improvement in treating this particular side effect in recent years (Todd and James 2008; Poltawski et al. 2008, 2009).

Troublesome though moderate sleepiness was found in 3.4% of our patients, which is a similar result to those of other studies (Homann et al. 2002; García Ruiz et al. 2008; Martinez-Martin et al. 2015; Dafsari et al. 2019). The same was true for episodes of mental confusion (9%), visual hallucinations (20.3%), and behavioral problems (5.3%) (Pietz et al. 1998; García Ruiz et al. 2008; van Laar et al. 2010).

Dyskinesias persisted in around 25% of patients, but only 3.3% found them problematic, which is comparable to other studies (Pfeiffer et al. 2007). Their continuing presence did not have a negative impact on either their quality of life or their impression of improvement.

The two groups did not differ on dropout frequency which, at 15% of patients, was lower than that reported in the literature after 6 months of treatment (approx. 30%) (Drapier et al. 2016; Sesar et al. 2017; Katzenschlager et al. 2021). As for the reasons given for dropping out, lack of efficacy and loss of motivation accounted for 45% of dropouts among our patients, while side effects, mainly neuropsychiatric, accounted for 30%, which is comparable to the data in the literature (Borgemeester et al. 2016; Drapier et al. 2016; Kimber et al. 2017; Sesar et al. 2017; Olivola et al. 2019; Henriksen and Staines 2021).

A total of four (2.5%) deaths were observed as follows: one in the hospital group, and three in the home group (*p* = 0.8). These occurred within the first few weeks of treatment for two of the patients, and during the fourth and fifth months for the two others. Three were among the seven patients who had severe difficulty swallowing. The cause of death was aspiration pneumonia for two of these patients, and choking for the third one. The frequency of occurrence was comparable to the rates reported in the literature: 2.5% at 6 months (Drapier et al. 2016) and 7.3% at 12 months (Sesar et al. 2017).

The different medical devices used to treat patients with PD (deep brain stimulation, chronic levodopa intestinal gel, and apomorphine pump) are all relatively expensive (Valldeoriola et al. 2013; Walter and Odin 2015), so it is important to try and reduce their cost without impacting quality of life. Although we found that home initiation required more visits by district nurses during the first month, patients became autonomous more quickly, meaning that the final number of visits was the same across the two groups. Moreover, although home initiation required two additional neurology consultations, this has to be set against a 7-day hospital stay. It is also worth pointing out that although home initiation required more visits by the device supplier (2.5 on average), this did not modify the cost, as the supplier charged a set fee. Home initiation requires greater commitment and more human resources from the supplier, but the latter could be recompensed in the medium and long term by greater patient satisfaction. Home initiation where the patient is supported by the device’s supplier and by a team of district nurses, supervised by the neurologist brings about a swifter improvement in the patient’s clinical status and quality of life and incurs a lower cost, as far fewer days are spent in hospital. However, the presence of a caregiver was an inclusion criterion. These results need to be confirmed in patients living without a caregiver.

The main limitation of this prospective study was the absence of randomization. The decision to embark on this treatment was made by the neurologist. Although patients were evenly distributed across the two groups at the beginning of the recruitment period, the difficulty of accessing routine or planned hospital care during the COVID-19 pandemic (Fabbri et al. 2021; Wan et al. 2022) induced a decrease of the recruitment in the hospital group and led some neurologists working in hospitals to favor home initiation. It should be noted that the imbalance that emerged between the two groups did not hinder our group comparisons. This further emphasizes the usefulness of the home initiation in terms of planning and delivering patient care (Roszmann et al. 2022).

## Conclusion

The present study demonstrated the efficacy and good tolerance of CSAI, regardless of initiation modality. Patients in the home group experienced a more rapid improvement in their quality of life and clinical status. Providing the treating neurologist has received the necessary training, and all the stakeholders (i.e., physician, patient, caregiver, nurse) are sufficiently motivated (Trenkwalder et al. 2015; Bhidayasiri et al. 2016), home initiation seems to bring satisfaction all round. The greater progressiveness of home titration seems to bring even more benefit. Many patients could therefore benefit from it, if they have the relevant medical profile (motor fluctuations, few or no cognitive disorders) (Henriksen 2014) and the right personal (motivation, understanding of the procedure and what it involves) and contextual (presence of a caregiver) characteristics. It is also important for neurologists to make themselves available during the initiation phase and to work closely with trusted suppliers, devoting the necessary time and energy to their patients’ care. The fact that home initiation is cheaper than in-hospital initiation means that CSAI should become more accessible to patients, with the treating neurologist playing a key role in patient follow-up. As neurologists working at a hospital or in private practice become more familiar with CSAI, they may start to prescribe it in more complex situations (difficulty swallowing, sleep problems, digestive surgery, palliative care) (Dewhurst et al. 2009; Auffret et al. 2022), to avoid sudden interruptions in treatment and the attendant complications, and improve patient comfort.

## Supplementary Information

Below is the link to the electronic supplementary material.

Supplementary file1 (DOCX 19 KB)

## Data Availability

The data that support the findings of this study are available from the corresponding author upon reasonable request.
