# Supplementary material for: Feasibility and benefits of home initiation of subcutaneous apomorphine infusion for patients with Parkinson’s disease: the APOKADO study
Source: J Neural Transm (Vienna). 2023 Mar 2;130(11):1463–74. doi: 10.1007/s00702-023-02609-6 (PMC9979890; doi:10.1007/s00702-023-02609-6)
Supplement: Supplementary file 1 — Supplementary file1 (DOCX 19 KB) [file 702_2023_2609_MOESM1_ESM.docx]

# Supplementary material:

# APOKADO study: Apomorphine set-up protocol

| DATE | | HOME | HOSPITAL | FLOW of APOMORPHINE | ORAL MEDICATION | VISITS |
| --- | --- | --- | --- | --- | --- | --- |
| D-8 | | Previsit by supplier, then training of DN and pharmacist |  |  | Unchanged | Clinical status, UPDRS, H&Y, MOCA, IADL, PDQ8 |
| D1 | | DN + Supplier | Hospital | 1 mg/hr | Unchanged |  |
| D2 | | DN + Supplier | Hospital | 1.5‑2 mg/hr | Unchanged |  |
| D3 | | DN | Hospital | 1.5‑3 mg/hr | Unchanged |  |
| D4 | | Supplier | Hospital | 2‑3 mg/hr | Unchanged |  |
| D5 | | DN | Hospital | 2‑3 mg/hr | Unchanged |  |
| D6 | | DN | Hospital | 2‑3 mg/hr | Unchanged |  |
| D7 | | DN | Hospital | 2‑3 mg/hr | Unchanged |  |
| D8 | | Neurologist | Neurologist  And Discharge | Depending on tolerance and efficacy | Adjustment |  |
| D11‑D12 | | Supplier: phone discussion with neurologist | Supplier: phone discussion with neurologist | Depending on tolerance and efficacy | Adjustment |  |
| D15 | | Neurologist | Neurologist | Depending on tolerance and efficacy | Adjustment |  |
| D18‑D19 | | Supplier: phone discussion with neurologist | Supplier: phone discussion with neurologist | Depending on tolerance and efficacy | Adjustment |  |
| D22 | | Neurologist | Neurologist | Depending on tolerance and efficacy | Adjustment |  |
| D25‑D26 | | Supplier: phone discussion with neurologist | Supplier: phone discussion with neurologist | Depending on tolerance and efficacy | Adjustment |  |
| M1 | | Neurologist | Neurologist | Depending on tolerance and efficacy | Adjustment | Clinical status, H&Y, PDQ8, CGI, autonomy, AE, costs |
| M3 | | Neurologist | Neurologist | Depending on tolerance and efficacy | Adjustment | Clinical status,H&Y, PDQ8,CGI, autonomy, AE, costs |
| M6 | | Neurologist | Neurologist | Depending on tolerance and efficacy | Adjustment | Clinical status, H&Y, PDQ8, CGI, autonomy, AE, costs |
|  | DN: district nurse; D: day; AE: adverse events; H&Y: Hoehn and Yahr score; CGI: Clinical Global Impression,  Mean recommended final flow rate: 4-7 mg/hr; bolus = 2-6 mg.  Increases in increments of 0.2‑0.5 mg/hr, depending on tolerance and efficacy.  *Notes:*  *When a dose adjustment seems necessary following a visit by the supplier (adjustment over the phone), the latter informs the neurologist, who then sends the patient a new prescription with the desired modification.*  *When the neurologist modifies the prescription, he/she informs the supplier as swiftly as possible, using the relevant channels.* | | | | |  |
